# Ginseng saponin metabolite 20(S)-protopanaxadiol inhibits tumor growth by targeting multiple cancer signaling pathways

**DOI:** 10.3892/or.2013.2438

**Published:** 2013-04-30

**Authors:** JIAN-LI GAO, GUI-YUAN LV, BAI-CHENG HE, BING-QIANG ZHANG, HONGYU ZHANG, NING WANG, CHONG-ZHI WANG, WEI DU, CHUN-SU YUAN, TONG-CHUAN HE

**Affiliations:** 1 Zhejiang Chinese Medical University, Hangzhou, Zhejiang 310053, P.R. China; 2 Molecular Oncology Laboratory, The University of Chicago Medical Center, Chicago, IL 60637, USA; 3 Department of Pharmacology and The First Affiliated Hospital, Chongqing Medical University, Chongqing 400016, P.R. China; 4 Department of Oncology, Southwest Hospital of The Third Military Medical University, Chongqing 400038, P.R. China; 5 Tang Center for Herbal Medicine Research, The University of Chicago Medical Center, Chicago, IL 60637, USA; 6 Ben May Department for Cancer Research, The University of Chicago Medical Center, Chicago, IL 60637, USA

**Keywords:** ginseng, ginseng metabolites, 20(S)-protopanaxadiol, colorectal cancer, signaling pathway, natural products

## Abstract

Plant-derived active constituents and their semi-synthetic or synthetic analogs have served as major sources of anticancer drugs. 20(S)-protopanaxadiol (PPD) is a metabolite of ginseng saponin of both American ginseng *(Panax quinquefolius* L.) and Asian ginseng (*Panax ginseng* C.A. Meyer). We previously demonstrated that ginsenoside Rg3, a glucoside precursor of PPD, exhibits anti-proliferative effects on HCT116 cells and reduces tumor size in a xenograft model. Our subsequent study indicated that PPD has more potent antitumor activity than that of Rg3 *in vitro* although the mechanism underlying the anticancer activity of PPD remains to be defined. Here, we investigated the mechanism underlying the anticancer activity of PPD in human cancer cells *in vitro* and *in vivo*. PPD was shown to inhibit growth and induce cell cycle arrest in HCT116 cells. The *in vivo* studies indicate that PPD inhibits xenograft tumor growth in athymic nude mice bearing HCT116 cells. The xenograft tumor size was significantly reduced when the animals were treated with PPD (30 mg/kg body weight) for 3 weeks. When the expression of previously identified Rg3 targets, A kinase (PRKA) anchor protein 8 (AKAP8L) and phosphatidylinositol transfer protein α (PITPNA), was analyzed, PPD was shown to inhibit the expression of PITPNA while upregulating AKAP8L expression in HCT116 cells. Pathway-specific reporter assays indicated that PPD effectively suppressed the NF-κB, JNK and MAPK/ERK signaling pathways. Taken together, our results suggest that the anticancer activity of PPD in colon cancer cells may be mediated through targeting NF-κB, JNK and MAPK/ERK signaling pathways, although the detailed mechanisms underlying the anticancer mode of PPD action need to be fully elucidated.

## Introduction

Plant-derived active constituents and their semi-synthetic or synthetic analogs have served as one of the major sources of anticancer drugs. An analysis of current chemotherapeutic agents and their sources indicates that nearly 60% of approved anticancer drugs are derived from natural products (1). Ginseng, such as American ginseng (*Panax quinquefolius* L.) and Asian ginseng (*Panax ginseng* C.A. Meyer), is the root of different *Panax* species (Araliaceae) and is one of the most commonly used traditional medicines. The saponins of ginseng (also known as ginsenosides) are its major active components and have been shown to possess anti-inflammatory, antitumor, and neuroprotective activities (2,3). Two types of ginsenosides in ginseng, protopanaxatriol (PTS) and protopanaxadiol (PDS) (2,4) have been shown to exert anticancer properties (5–9). After oral administration of PDS ginsenosides (e.g., Rg3) to mice, PDS is metabolically converted to protopanaxadiol (PPD) and Compound K (CK) by intestinal bacteria (10,11). Compound K can significantly inhibit the PMA-induced MMP-9 secretion and protein expression via suppressing the DNA-binding and transcriptional activities of AP-1, which is the downstream factor of p38 MAPK, ERK and JNK (12). Thus, it is of importance to understand the anticancer effects and possible mechanisms associated with ginseng derivatives.

We previously investigated the cancer chemopreventive activities of American ginseng root extracts (AGE and S-AGE), fractions (S2h) and pure ginsenoside Rg3 on human colorectal cancer cells (13). Ginsenoside Rg3 was shown to exert antiproliferative effects on HCT116 cells *in vitro* and to inhibit tumor growth in a nude mouse xenograft tumor model (14). Furthermore, we conducted a microarray expression profiling analysis and found that the expression levels of 76 genes, such as A kinase (PRKA) anchor protein 8 (AKAP8L) and phosphatidylinositol transfer protein α (PITPNA), were differentially regulated after the treatment of HCT116 cells with S2h (American ginseng extract) or ginsenoside Rg3 (13). As one of the most important metabolites of the ginseng herb, PPD and its derivates have therapeutic potential for inhibiting the growth and invasiveness of tumors. However, the molecular mechanisms underlying the anticancer activity of PPD remain to be fully elucidated.

The present study investigated the anticancer effects of PPD and its mode of action in human cancer cells. We found that PPD inhibited growth and induced cell cycle arrest in HCT116 cells. Furthermore, PPD inhibited the xenograft tumor growth in athymic nude mice. The xenograft tumor size was significantly reduced following treatment with PPD for up to 3 weeks. Furthermore, PPD inhibited the expression of PITPNA while upregulating AKAP8L expression in HCT116 cells. Pathway-specific reporter assays indicated that PPD effectively inhibited the NF-κB, JNK and MAPK/ERK signaling pathways. Thus, our results suggest that PPD may exert its anticancer activity on colon cancer cells through targeting major signaling pathways, such as NF-κB, JNK and MAPK/ERK.

## Materials and methods

### Chemicals and drug preparations

PPD was kindly provided by Professor Ping Li of China Pharmaceutical University (Nanjing, China) with a purity >95% as confirmed by HPLC (4,15). PPD was dissolved in dimethyl sulfoxide (DMSO) (15 mM stock solution). For *in vivo* treatment, PPD was dissolved in PEG. Unless otherwise indicated, all chemicals were obtained from Fisher Scientific (Pittsburgh, PA, USA) or Sigma-Aldrich (St. Louis, MO, USA).

### Cell culture

Human colorectal cancer lines (HCT116 and SW480), breast cancer cell lines (MDA-MB-468 and MDA-MB-231), prostate cancer cell lines (PC3 and DU145), osteosarcoma cell lines (MG63 and 143B) and HEK-293 cells were purchased from the American Type Culture Collection (ATCC, Manassas, VA, USA) and grown in Dulbecco’s modified Eagle’s medium (DMEM) (Invitrogen Life Technologies, Carlsbad, CA, USA) supplemented with 10% fetal bovine serum (FBS; HyClone Laboratories, Logan, UT, USA) and 50 units penicillin/streptomycin in 5% CO_2_ at 37°C.

### MTT proliferation assay

A modified MTT assay was used to examine the cell growth inhibitory effect of ginsenosides on cell proliferation as previously described (16). Cells were seeded in 96-well plates (1×10^4^ cells/well, 50–70% density). Ginsenosides were added to the cells at various concentrations and incubation was carried out for 48 h. Fifteen microliters of dye solution was added to each well and incubated for an additional 4 h. One hundred microliters/well solubilization/stop solution was added to stop the reaction and to solubilize the formazan crystals in a humidified atmosphere overnight. Absorbance at 570 nm was determined using a 96-well microplate reader.

### Crystal violet assay

HCT116 cells were treated with the indicated concentrations of drugs. At the endpoints, the cell culture medium was carefully removed. The wells were gently washed with phosphate-buffered saline (PBS) at room temperature. The medium was aspirated and cells were stained with 0.5% crystal violet formalin solution at room temperature for 20–30 min. After staining, the cells were washed with tap water and air-dried at room temperature (17,18).

### Flow cytometry and cell cycle analysis

Flow cytometry was carried out to quantitatively detect the cell cycle distribution (19). Cells were plated into 6-well plates for drug treatments. At 24, 48 and 72 h post treatment, cells were harvested, washed with PBS, fixed in cold methanol overnight at 4°C and stained with 50 ng/ml propidium iodide (PI) by incubation at 4°C for 15 min. The stained cells were analyzed by flow cytometry.

### RNA isolation and semi-quantitative reverse transcription-polymerase chain reaction (RT-PCR) analysis

Total RNA was isolated using TRIzol reagents and used to generate cDNA templates by RT reaction with hexamer and SuperScript^®^ II RT (both from Invitrogen Life Technologies). The first strand cDNA products were further diluted 10-fold and used as PCR templates. Semi-quantitative RT-PCR was carried out as described (20). Briefly, PCR primers were designed using the Primer3 program to amplify the human genes of interest (product sizes 150–180 bp) as follows: GAPDH forward, 5′-CAACGAATTTGGCTACAGCA-3 and reverse, 5′-AGGGGAGATTCAGTGTGGTG-3′; PITPNA forward, 5′-CGTCCTACCCCCATGTTG-3′ and reverse, 5′-ACTGGGCAGCGTCTGTTC-3′; and AKAP8L forward, 5′-GCAGGCAGGCAAGAAGAG-3′ and reverse, 5′-TGGCCATCTCGTCCTCAT-3′. A touchdown cycling program was carried out as follows: 94°C for 2 min for 1 cycle, 92°C for 20 sec, 68°C for 30 sec, and 72°C for 12 cycles with a decrease of 18°C per cycle and then at 92°C for 20 sec, 57°C for 30 sec, and 72°C for 20 sec for 20 to 25 cycles depending on the abundance of a given gene. The specificity of PCR products was confirmed by resolving PCR products on 1.5% agarose gels. All samples were normalized with the internal control GAPDH.

### Xenograft tumor model and xenogen bioluminescence imaging

The HCT116-Luc cell line, which stably expresses firefly luciferase, was generated as previously described (19,21). Animal use and care were carried out according to the protocol guidelines approved by the Institutional Animal Care and Use Committee. Athymic nude mice (female, 4–6 weeks old, ~20 g body weight, n=5/group; Harlan SD, Indianapolis, IN, USA) were used. HCT116-Luc cells were harvested and resuspended in PBS to a final density of 2×10^7^ cells/ml. Cells (1×10^6^) were injected subcutaneously into the flanks of the mice. At 1 week post injection, PPD was administered (30 mg/kg) through an i.p. injection once every 2 days for 3 week.

For whole body bioluminescence imaging, animals were anesthetized with isoflurane attached to a nose-cone mask within the Xenogen IVIS 200 imaging system after implantion for 1 week. Mice were injected (i.p.) with D-Luciferin sodium salt (Gold BioTechnology, St. Louis, MO, USA) at 100 mg/kg in 0.1 ml sterile PBS. The pseudo-images were obtained by superimposing the emitted light over the grayscale images of the animal. Quantitative analysis was carried out with Xenogen’s Living Image V2.50.1 software as described (13,19). Animals were sacrificed after 3 weeks, and tumor samples were retrieved for histologic examination.

### Histologic evaluation and immunohistochemical staining

Retrieved tumor tissues were fixed in 10% formalin and embedded in paraffin. Serial sections of the embedded specimens were stained with hematoxylin and eosin. Paraffin-embedded sections were deparaffinized and then rehydrated in a graduated manner. The deparaffinized samples were subjected to antigen retrieval and fixation. Slides were blocked and probed with an antiproliferating cell nuclear antigen (PCNA) antibody (Santa Cruz Biotechnology, Inc., Santa Cruz, CA, USA), followed by incubation with the anti-mouse IgG-biotin secondary antibody. Finally, sections were incubated with HRP-streptavidin and visualized by 3,3′-diaminobenzidine staining (22).

### Construction of pathway-specific Gaussia luciferase reporters, establishment of HCT116-GLuc reporter lines, and Gaussia luciferase assay

Promoters responsive to the following signaling pathways, including MAPK/ERK, MAPK/JNK, Wnt, Notch, cell cycle/pRb-E2F, NF-κB, Myc/Max, hypoxia (namely Elk-1/SRF, AP-1, TCF/LEF, RBP-Jκ, E2F/DP1, NF-κB and hypoxia-inducible factor-1) were cloned into our homemade pBGLuc retroviral vector. All subcloned promoter fragments were verified by DNA sequencing.

Stable HCT116-GLuc reporter lines were established using the retroviral transduction approach as previously described (14,19,21). Gaussia luciferase activity was determined using the Gaussia luciferase assay kit (New England Biolabs). Briefly, HCT116-GLuc cell lines were seeded in 24-well culture plates and treated with 0 or 10 μM PPD. After 24 h, cell culture medium was subjected to Gaussia luciferase assay. Each assay condition was conducted in triplicate.

### Statistical analysis

The *in vitro* experiments were performed in triplicate. Data are expressed as the means ± standard error (SE). Statistical significances between vehicle treatment vs. drug-treatment were determined by one-way ANOVA and the Student’s t-test. A value of p<0.05 was considered to indicate a statistically significant result.

## Results

### PPD inhibits the proliferative activity of human cancer cells in vitro

**Figure 1:**
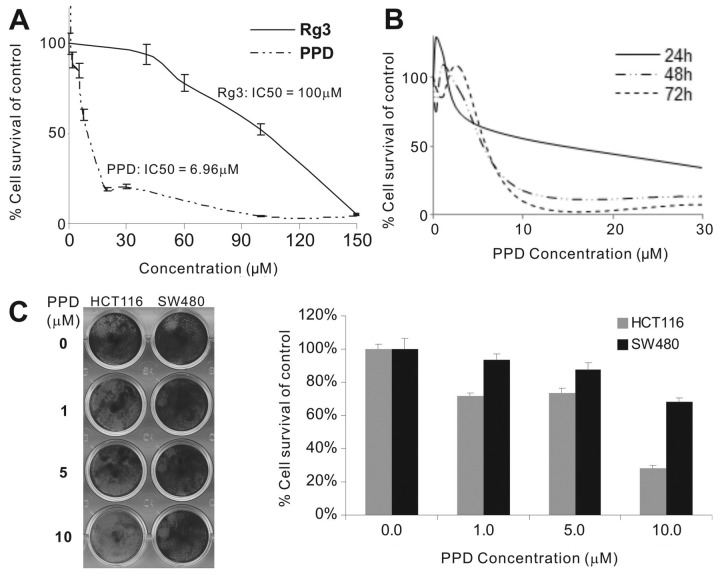
Effect of protopanaxadiol (PPD) on the proliferation of human cancer cells. (A) MTT assay. HCT116 cells were seeded in 24-well plates and treated with different concentrations of PPD and Rg3 for 48 h. Cells were fixed and subjected to MTT assay. Each treatment condition was carried out in triplicate. (B) Crystal violet assay. HCT116 cells were treated with PPD at the indicated concentrations for 24, 48 and 72 h. Treated cells were subjected to crystal violet staining, which was subsequently dissolved for quantitative readings. Each assay condition was carried out in triplicate. (C) Crystal violet assay in HCT116 and SW480 cell lines. HCT116 and SW480 cells were treated with PPD at the indicated concentrations for 72 h. The gross images (left panel) and quantitative analysis (right panel) of crystal violet staining were obtained. Each assay condition was calculated in triplicate.

The effect of PPD and ginsenoside Rg3 on the proliferation of HCT116 cells was evaluated by MTT assay. The IC_50_ of PPD was significantly lower than that of Rg3 in the HCT116 cells (Fig. 1A). At 10–30 μM, PPD exhibited strong anti-proliferation effects after 48 and 72 h of treatment (Fig. 1B). We also investigated whether PPD inhibits the viability of other human cancer cell lines. Treatment of different types of cancer cells with different dosages of PPD for 48 h significantly suppressed the cell proliferation of the tested cancer cell lines: human colon cancer (HCT116 and SW480), breast cancer (MDA-MB-468 and MDA-MB-231), prostate cancer (PC3 and DU145) and osteosarcoma cell lines (MG63 and 143B) (data not shown). The IC_50_ value for PPD in these cancer cell lines was 4.69 μM for HCT116, 8.99 μM for SW480, 7.64 μM for MDA-MB-468, 4.49 μM for MDA-MB-231, 1.40 μM for PC3, 4.71 μM for DU145, 5.17 μM for MG63, and 8.36 μM for 143B, respectively. Crystal violet staining assay revealed that 10 μM PPD had anti-proliferation effects on the HCT116 cells (cell viability <30%) although PPD was less effective on the SW480 cells (Fig. 1C), which was consistent with the differences in their IC_50_ values.

### PPD restricts the proliferating cancer cells in the G1/S phases of the cell cycle

**Figure 2:**
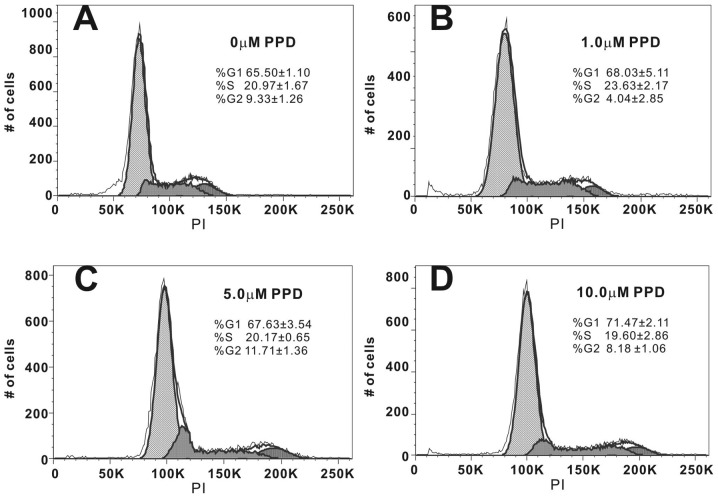
Cell cycle analysis of protopanaxadiol (PPD)-treated cells. Subconfluent HCT116 cells were treated with (A) 0, (B) 1, (C) 5 and (D) 10 μM PPD for 48 h. Cells were fixed, labeled with propidium iodide (PI), and subjected to flow cytometry. Histograms indicating the DNA content (x-axis, PI-fluorescence) vs. cell count (y-axis) are shown.

In order to better understand the mechanism behind PPD-mediated inhibition of cell proliferation, we analyzed the distribution of PPD-treated cancer cells in different phases of the cell cycle by flow cytometry following treatment of cells with different concentrations of PPD for 48 h. We found that PPD caused a dose-dependent cell accumulation in the G1/S phase (Fig. 2). For example, treatment of HCT116 cells with 10 μM PPD led to an 86 to 91% increase in cells in the G1+S phase (Fig. 2A vs. D), leading to fewer cells progressing to the G2 phase. These results indicate that cell cycle progression was significantly blocked in the G1/S phase when cells were treated with PPD.

### PPD effectively inhibits tumor growth in vivo

**Figure 3:**
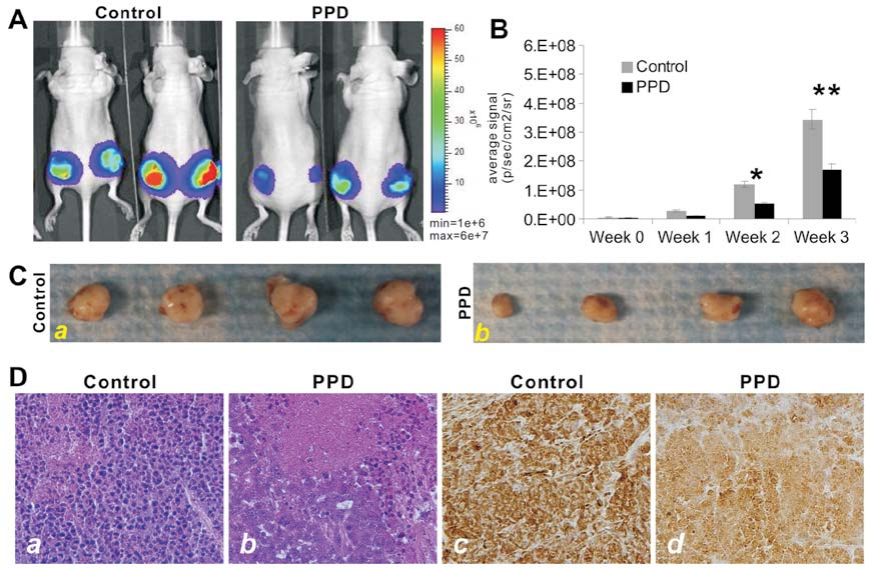
Anticancer effect of protopanaxadiol (PPD) on HCT116 tumor-bearing nude mice. (A) Xenogen bioluminescence imaging. Firefly luciferase-tagged HCT116 cells were subcutaneously injected into the flanks of athymic nude mice. At 1 week post tumor cell injection (i.e., week 0), PPD was administered (30 mg/kg) by i.p. injection once every 2 days for 3 week. Xenogen imaging was conducted weekly. Representative images of the control and PPD treatment groups at week 3 post treatment are shown. (B) Xenogen bioluminescence imaging analysis. The obtained Xenogen imaging signal intensity (photons/sec/cm^2^/steradian) at 1, 2 and 3 weeks post treatment with PPD were quantitatively analyzed. ^*^p<0.05; ^**^p<0.01. (C) Representative gross images of the retrieved xenograft tumors at the endpoint (3 week post treatment). (D) Histologic and proliferation analyses of the retrieved samples. The samples were fixed, paraffin-embedded, sectioned, and subjected to hematoxylin and eosin (H&E) staining (a and b). For cell proliferation analysis, the sections were subjected to immunohistochemical staining with an anti-PCNA antibody (c and d). Representative results are shown.

We further tested the antitumor activity of PPD in a xenograft tumor model of human colon cancer. The firefly luciferase-tagged HCT116 cells were subcutaneously injected in mice to form xenograft tumors. At one week, the tumor-bearing athymic nude mice were i.p. administered PPD at 30 mg/kg once every 2 days for up to 3 weeks. The tumor growth was monitored by using whole body Xenogen imaging (Fig. 3A). PPD was shown to significantly inhibit xenograft tumor growth at 2 weeks after treatment (Fig. 3B). At the endpoint (3 months after treatment), the xenograft tumors were retrieved and were shown to be smaller in the PPD treatment group (Fig. 3C). Histologically, the PPD-treated xenograft tumors exhibited significant necrosis (Fig. 3D-a vs. -b). Immunohistochemical staining with a PCNA antibody revealed that xenograft tumor cells treated with PPD exhibited a marked decrease in cell proliferation (Fig. 3D-a vs. -b). Thus, the *in vivo* results suggest that PPD may be developed into an efficacious anticancer agent.

### Rg3 targets AKAP8L and PITPNA may be involved in the antitumor effect of PPD

**Figure 4:**
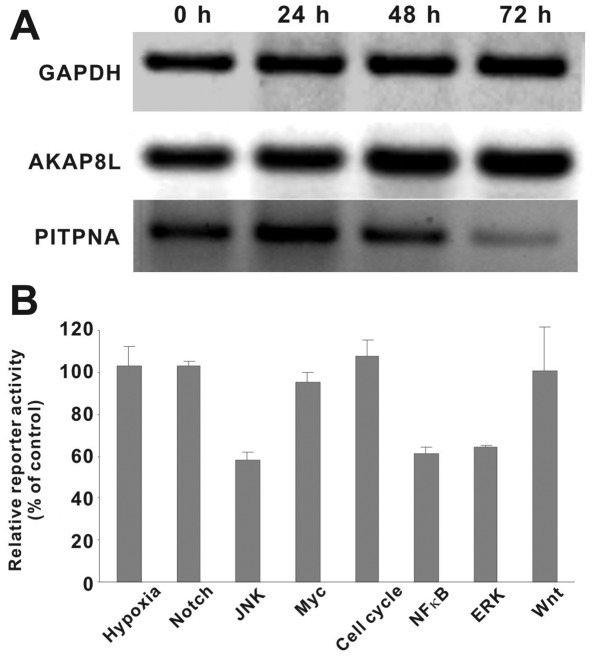
Protopanaxadiol (PPD) targets multiple signaling pathways. (A) The expression of anchor protein 8 (AKAP8L) and phosphatidylinositol transfer protein α (PITPNA) in PPD-treated cancer cells. Subconfluent HCT116 cells were treated with PPD (10 μM). Total RNA was collected at the indicated time points and subjected to semi-quantitative reverse transcription-polymerase chain reaction (RT-PCR) analysis with primer pairs for human GAPDH, AKAP8L and PITPNA transcripts. Representative results are shown. (B) The effect of PPD on 8 different signaling pathways in HCT116 cells. Various subconfluent HCT116-GLuc cells lines were seeded in 24-well plates and treated without or with 10 μM PPD. At 24 h, cell culture medium was subjected to Gaussia luciferase assay. Each assay condition was carried out in triplicate.

We previously found that the expression levels of AKAP8L and PITPNA were significantly altered following treatment with S2h (American ginseng extract) or ginsenoside Rg3 in HCT116 cells (13). As one of the main metabolites of S2h and the aglycon of ginsenoside Rg3, PPD was shown to affect the expression levels of AKAP8L and PITPNA in the PPD-treated HCT116 cells (Fig. 4A). Specifically, PPD treatment slightly upregulated AKAP8L expression while significantly inhibiting PITPNA expression in a time-dependent fashion. These results suggest that upregulation of AKAP8L and/or downregulation of PITPNA may play an important role in mediating the anticancer activities conferred by ginsenoside derivatives, such as PPD. However, the exact mechanism behind their roles in the anticancer action of PPD needs to be thoroughly investigated.

### PPD targets MAPK and NF-κB signaling pathways in human colon cancer

We sought to further investigate the mechanistic basis underlying the anticancer activity of PPD. Although the above results indicate that Rg3 targets AKAP8L and PITPNA may play an important role in the mode of anticancer action of PPD, it is known that cancer development usually hijacks multiple cellular signaling pathways (23,24). Thus, we tested the effect of PPD on 8 major cancer-related signaling pathways, including MAPK/ERK, MAPK/JNK, Wnt, Notch, cell cycle/pRb-E2F, NF-κB, Myc/Max and hypoxia. When HCT116 cells containing the pathway-speciifc reporters were treated with 10 μM PPD, we found that the relative reporter activities for NF-κB, MAPK/JNK and MAPK/ERK pathways were significantly inihibited (Fig. 4B). The other 5 pathways, noticeably Myc/Max and Wnt, were not significantly affected by PPD in HCT116 cells. Thus, these results suggest that PPD may exert its anticancer activity at least in part by targeting the ERK, JNK and/or NF-κB signaling pathways although the exact mechanism needs to be fully elucidated.

## Discussion

In the present study, we demonstrated the effectiveness of PPD, a metabolite of ginseng saponin, against multiple tumor types in human cancer cell culture and animal models. PPD inhibited human cancer cell growth in 8 types of human cancer cells and these results were consistent with other reports on the effects of PPD on human cancer cell lines (25–29). Previous studies have shown that PPD induced cell cycle arrest in the G0–G1 phase in human hepatocellular carcinoma SMMC7721 cells (30) or in the G1 phase in U937 human monocytic leukemia cells (31). Similar variability was observed in the HCT116 colon cancer cells.

Ginseng is one of the most widely used medicinal plants and remains a top selling natural product globally. The major bioactive constituents in ginseng are ginsenosides, a group of triterpene glycosides (2,4). Several natural ginseng saponins have been shown to exhibit high potency against multiple tumor types in cell culture and animal models (32). Kim *et al*(33) studied 11 ginsenosides and determined that Rg3 and Rh2 inhibited the proliferation of prostate cancer cells. Iishi *et al*(34) used a rat AOM-induced tumor model to determine the effects of Rg3 in inhibiting the cell proliferation of colon cancer cells. Jia *et al*(35) reported that Rh2 inhibited proliferation, induced apoptosis in cancer cell lines, and sensitized drug-resistant breast cancer cells to paclitaxel. Rk1 and Rg5 were also found to inhibit the proliferation of human hepatocellular carcinoma cells (36,37).

Notably, Compound K [(20-*O*-(β-D-glucopyranosyl)-20(S)-protopanaxadiol)], one of the most important intestinal metabolites isolated from ginseng PDS saponins, can also induce apoptotic cell death concurrent with cell cycle arrest in the G0–G1 phase in SMMC7721 cells (30) and G1 phase arrest in U937 cells (31). Compound K was also found to inhibit the cell viability and induce apoptosis of human gastric carcinoma cells via the Bid-mediated mitochondrial pathway (38). Moreover, Compound K significantly inhibited PMA-induced MMP-9 secretion and protein expression by suppressing DNA-binding and transcriptional activities of AP-1, which is the downstream factor of p38 MAPK, ERK and JNK (12). Similar to many other herbal medicines, ginseng is usually taken orally. In this form its bioavailability is low due to incomplete absorption (39). To date, the biotransformation of ginsenosides to their metabolites by intestinal bacteria has been reported. Some of the metabolites, such as Compound K and PPD, have shown various bioactivities including cancer chemoprevention (15). Nonetheless, the anticancer mechanisms of these ginseng metabolites are largely unknown.

The low *in vivo* toxicity of PPD suggests that this compound or its derivatives may have potential for clinical applications in cancer chemotherapy (2). However, PPD is a highly hydrophobic molecule with limited water solubility and has low *in vivo* uptake. In our *in vivo* studies, we used PEG and PEG400 to improve the solubility of PPD. Our results clearly demonstrated that this formulation could circumvent the limited solubility and/or bioavailability of PPD.

The glycoside of PPD was shown to induce apoptosis of human prostate cancer cells via inhibition of the NF-κB (40,41), JNK and ERK pathways (12). PPD may induce apoptosis by decreasing caspase-3 activity (25,42), MMP secretion (26) or the ER stress pathway (27). However, it remains unclear how PPD targets cancer-related signaling pathways. In the present study, we used a cell-based, unbiased, pathway-specific analysis and identified three major pathways that may be targeted by PPD. Thus, future investigation should be directed towards how these pathways are inhibited. These lines of investigations are critical for the potential clinical use of PPD as an anticancer agent.

In conclusion, we demonstrated that PPD effectively inhibits cell proliferation and tumor growth of human cancer both *in vitro* and *in vivo*. We demonstrated that the anticancer activity of PPD in colon cancer cells is at least in part due to the downregulation of multiple signaling pathways, noticeably MAPK/ERK, MAPK/JNK and NF-κB. Although further investigations are required to dissect the underlying mechanisms, these results illustrate the potential clinical applications for PPD alone or in combination with other anticancer agents.
